# Precision Medicine in Solid Tumors: How Far We Traveled So Far?

**DOI:** 10.3390/cancers14133202

**Published:** 2022-06-30

**Authors:** Nandini Dey, Pradip De

**Affiliations:** 1 Translational Oncology Laboratory, Avera Research Institute, Sioux Falls, SD 57105, USA; pradip.de@avera.org; 2 Department of Internal Medicine, University of South Dakota SSOM, USD, Sioux Falls, SD 57105, USA

The future of disease management in solid tumors will rely heavily on how effectively we understand precision medicine and how successfully we can deliver personalized medicine. In the post-human genome project era, both translational research as well as clinical care in oncology has become functions of knowledge-based deliverance of therapy. The knowledge rides on the technological revolution, next-generation sequencing (NGS), and whole-exome sequencing/whole transcriptome sequencing (WES/WTS), which provide comprehensive genomic data in real-time from the tumor, tumor- microenvironment (TME), and blood. The wealth of information help clinicians interrogate the genomics-driven disease and fuels the decision-making in precision medicine.

During the inception of this Special Issue, entitled “Precision Medicine in Solid Tumors”, we promised to present an in-depth review of the topic’s current status. We covered (A) the challenges of NGS and WES/WTS in reaching a saturation point for finding a new effective target in oncology; (B) the holistic aspect of tumor biology from the viewpoint of tumor-TME-liquid biopsy; (C) mutation-guided treatment; (D) the enormity and legality of the data, electronic medical record; and (E) the translation of knowledge to patient outcomes and clinical guidelines. The Special Issue presents 11 original research articles, 2 review articles, 1 opinion, and 2 brief reports.

## 1. NGS & WES/WTS

Precision medicine seeks to use genomic data (alteration, such as mutations, amplifications, copy number variations, chromosomal rearrangements) to help provide the right treatment to the right patient at the right time. In the last 15 years since the invention of this breakthrough technology, NGS technology provided the genetic constitution of different types of cancers. The speed, accuracy, and cost affordability of NGS have helped spur the advent of precision medicine, which involves designing a treatment based on disease-driving molecular alterations [1,2] (Collins F. Precision Oncology: Gene changes predict immunotherapy response (NIH Director’s Blog; accessed on 10 November 2017)). In today’s world, WES/WTS integrates tumor-normal matched samples. It offers one comprehensive test to rapidly deliver in-depth (>18,000 genes) molecular insight and avoid running multiple sequential panels to unlock the answer to a patient’s cancer. WES/WTS-driven comprehensive molecular analysis has identified a relatively high incidence of potentially targetable genomic alterations in solid tumors, predictive of response to targeted and immunotherapies. NGS and tumor mutation profiling have become essential diagnostic/decision-making tools for routine use in oncology clinics, including community-based clinics. In a retrospective study, Inagaki et al. tested the clinical utility of NGS-based panels in the Universal Health-Care System in Japan from a single University hospital in Osaka and reported that the NGS assay should be performed earlier in the clinical course to maximize the clinical benefit. The study revealed that the broader reimbursement for the NGS assay would enhance the delivery of precision oncology to patients. Heong et al. tested the feasibility of a “Multi-Regional” sample biopsy from metastatic lesions to evaluate actionable truncal mutations using a Single-Pass Percutaneous Technique by WES. They demonstrated the strength of their evaluation in prioritizing precision-therapy strategies. In debating the implication of NGS in a laboratory setting versus in real-world clinical practice, Singh et al. presented the impact and diagnostic gaps of comprehensive genomic profiling in a study participated by the University of Pennsylvania/Abramson Cancer Center, PA, USA, NYU Langone Perlmutter Cancer Center, New York, USA, and Montefiore Medical Center/Albert Einstein College of Medicine, Bronx NY, USA. Their study concluded that routine use of CGP in the community across all cancer types detects potentially actionable genomic alterations in most patients. In Silico Simulation of targeted gene panels is a powerful tool for the development of technology. Noskova et al. presented a study that evaluated TMB in multiple pediatric tumors by Real-Life Whole-Exome Sequencing and In Silico Simulation of two major targeted gene panels to evaluate the choice of method which affect the clinical decision. Their study confirmed a significant technological variability introduced by different laboratory techniques and various settings of bioinformatics pipelines.

## 2. Tumor-TME-Blood

Transformed tumor cells reside within their non-transformed host-microenvironment. With the advent of advanced technology to pinpoint both cellular and acellular characteristics of a tumor mass, the relationship between tumor cells and their non-transformed microenvironment has been acknowledged [3,4]. The acknowledgment has come from the translational and clinical research indicating holistic support of TME to tumor cells during the progression of the disease [5,6] by influencing tumor growth, formation of stem cell niches, immunosuppression, metastasis, and drug resistance. The TME encompasses both cellular components, the extracellular space containing both soluble cytokines and insoluble extracellular matrix (ECM) components. The recognition of the undeniable consequence of the ‘unholy alliance’ of the neoplastic tumor cells and their inherently dynamic non-neoplastic components of the microenvironment [7,8] has led to the incorporation of targeting TME for cancer treatments, including immunotherapy and radiotherapy in recent years [9,10,11]. As the interaction between the tumor and its TME evolves in a complex bidirectional manner, there was a long-lasting search for finding a “mirror room” that could serve as a surrogate of the actual events at the tumor site. In the last decades, the search has revealed a source-easy sample that can be the “mirror room” for the tumor-TME events in peripheral blood (liquid biopsy). Circulating tumor cells (CTC), ctDNA, cancer-associated fibroblasts (CAF), cell fusions, CAMLS, immune cells, exosomes, soluble proteins (sPD-L1, sPD-L2, sPD-1) from the blood have been beginning to show the reflection of the tumor-TME events about cancer screening, early detection, drug effect, on-treatment monitoring, drug resistance and post-treatment surveillance [12,13,14,15,16]. Burcher et al. demonstrated the prevalence of DNA repair gene mutations in blood and tumor tissue and their impact on prognosis and treatment in HNSCC. A single-institution retrospective study was undertaken to test the profiles of 170 patients with HNSCC and available tumor tissue DNA (tDNA) and circulating tumor DNA (ctDNA). Results were analyzed for mutations in a set of 18 DDR genes as well as in gene subsets defined by technical and clinical significance. This study presents the largest cohort to date to analyze the genomic landscape in both blood and tumor tissue in patients with HNSCC and reports a high prevalence of DDR gene mutations in this tumor type. Patients with DDR gene mutations in ctDNA rather than tDNA had shown significantly worse prognoses, with a more advanced disease burden at the end of the study and with decreased overall survival. Sulaiman et al. provided a method for a user-friendly and cost-effective detection of CTC. The technique’s power can be tested as a single-point at the baseline during surgery and in a multi-point longitudinal mode during and after a treatment regimen. To this end, studies showed that meaningful information could be obtained from patients’ plasma, offering an avenue for longitudinal surveillance during treatment and post-treatment monitoring period. In a brief report, Shin et al. presented a highly sensitive NGS-based genotyping platform for EGFR mutations in plasma from NSCLC patients. Their study demonstrated that Sel-Cap is a highly sensitive platform for EGFR mutations in plasma, and the timing of the first appearance of T790M mutation in plasma, determined via highly sensitive liquid biopsies, may be useful for the prediction of disease progression of NSCLC around five months in advance. Similarly, Kim et al. evaluated 2 EGFR mutation tests on tumors and plasma from patients with NSCLC. The study reported the interchangeable use of two EGFR mutation tests, cobas v2 and PANAMutyper, in tumor and plasma EGFR testing. Both tests in their study have high diagnostic precision in plasma but are particularly valuable in late-stage disease. Their clinical data in T790M carriers strongly support the clinical benefits of osimertinib treatment guided by both EGFR mutation tests. De et al. interrogated the role of TME in the development of resistance to chemotherapy and targeted therapy. Cancer-Associated Fibroblasts (CAFs) are one of the components of the TME that is used by tumor cells to achieve resistance to therapy. Their review interrogated the irrefutable role of CAFs in the development of resistance that would strategize the ability to design improved therapies inclusive of CAFs in light of currently ongoing and completed CAF-based NIH clinical trials.

## 3. Mutation-Guided Treatment-ICI Therapy

Since genomic alteration(s) and chromosomal instability are the primary determinants of cells that acquire malignant traits, a cancer-specific genomic map provides the roadmap for the treatment. This treatment philosophy is state-of-art in today’s clinics and is called precision oncology, which embraces clinical decisions based on genomic/proteomic data. Today’s success in treatment modalities and overall management of cancer, both pathway-targeted and immune-targeted therapy, are empowered by mutation-guided target-specific drugs [17]. Tumors have been known to adopt and bypass the PD-1/PD-L1 axis to achieve immune evasion, and the PD-1/PD-L1 axis has been accepted as an obvious target treated by immune checkpoint inhibitors (ICI). On this basis, PD-L1 protein expression on tumor or immune cells emerged as the first potential predictive biomarker for sensitivity to immune checkpoint blockade. In 2015, PD-L1 was the first FDA-approved predictive biomarker for non-small-cell lung cancer (NSCLC) [18]. Nine FDA approvals have been linked to a specific PD-L1 threshold and companion diagnostics, including bladder cancer (N = 3), non-small cell lung cancer (N = 3), triple-negative breast cancer (N = 1), cervical cancer (N = 1), and gastric/gastroesophageal junction cancer (N = 1) out of which 88.9% have been targeted with ICI monotherapy [19]. Following the IMpower110 (NCT02409342) clinical trial (in May 2020), the inclusion criteria of high PD-L1 expression ≥50% of tumor cells or ≥10% of tumor-infiltrating immune cells (as defined by an FDA-approved device) were FDA approved for the treatment of adult metastatic NSCLC with no EGFR or ALK genomic aberrations [20]. In the following month, the FDA expanded the approval of pembrolizumab (PD-1 inhibitor), routinely used as immunotherapy in a variety of cancer patients) to include unresectable or metastatic tumors with TMB-H (≥10 mutation/Mb) that have progressed following prior treatment with no satisfactory alternative therapy options, based on the Keynote-158 study (NCT02628067) [21]. Currently, FDA has approved 3 predictive biomarkers, including PD-L1, microsatellite instability (MSI), and tumor mutational burden (TMB), including blood-TMB for patient selection for ICI response in clinical practice. Burcher et al. studied the relationship between TMB, PD-L1, patient characteristics, and response to ICI in HNSCC. Their work demonstrated the utility of TMB as a prognostic variable and predictive marker of response to ICI. The study also pointed to the significant association of high TMB with active tobacco use and primary tumor location in the larynx. In their study, high PD-L1 values were associated with the African American race, high T stage, high overall disease stage, non-/ex-smokers, and non-/ex-drinkers. Higuchi et al. study primary driver mutations in GTF2I specific to the development of thymomas. Their study showed that the majority of thymomas harbor mutations in GTF2I that can be potentially used as a novel therapeutic target in patients with thymomas. Tamara Ius et al. from Italy presented a novel comprehensive clinical stratification model to refine prognosis in GBM. Their prognostic score uses clinical/molecular and images data that can be useful to stratify GBM patients undergoing surgical resection. By using the random forest approach [CART analysis (classification and regression tree)] on Survival time data of 465 cases, they developed a new prediction score resulting in 10 groups based on the extent of resection (EOR), age, volumetric tumor features, intraoperative protocols, and molecular tumor classes. Their score could be helpful in a clinical setting to refine the prognosis of GBM patients after surgery and before postoperative treatment. Hossain et al. discussed tumor heterogeneity and sub-clonal evolution in primary and metastatic TNBC, which still remains a challenge for oncologists to design adaptive precision medicine-based treatment plans.

## 4. Electronic Medical Record

In today’s clinical world, electronic data recording, management, and safety are as important as any branches of disease care. One of the reasons for this is that the Electronic Medical Record (EMR) is viewed as a solution to many of the shortcomings of health care systems, and therefore, its importance is realized to improve patient care [22]. The importance of the electronic health record (EHR) system is highlighted by the promise of substantial benefits, including better patient care and decreased healthcare costs, useability and accessibility of records in one hand, while the poor EHR system design with improper implementation invites EHR-related errors jeopardizing the integrity of the information in the EHR, leading to errors that endanger patients safety or decrease the quality of care and serious unintended consequences in another hand [23]. A limited EMR is often preferred to a faulty EMR from the patients’ safety point of view [24,25,26]. The future will prove the feasibility of a collaborative, noteless EMR design with minimum information chaos, the highest level of patient data protection, and a user-friendly operation for managing team workflows at the clinics [27]. Jibiki et al. investigated a case of Memorial Sloan Kettering-Integrated Mutation Profiling of Actionable Cancer Targets (MSK-IMPACT), a tumor profiling test approved by the U.S. FDA in 2017, to examine what factors would contribute to healthcare innovation. Their study conducted comparative analyses of three tumor profiling tests approved by the U.S. FDA in 2017, hypothesizing that the FDA’s regulatory reforms, early application of new technologies to both research and clinical settings, and open data accumulated as a result of large-scale research programs have promoted new drug development in oncology. The study set three parameters to observe cases. First, the FDA regulatory reforms. Second, early application of new technologies, such as NGS, to both research and clinical settings. The third is the accumulation of open data. The study discussed the implications potentially suggested by the outcomes and challenges of the three cases. Brown et al. presented the opinion on the use of EMR to identify potentially eligible study subjects for lung cancer screening with biomarkers which explores the current issues in and approaches to lung cancer screening and whether records can be used to identify eligible subjects for screening and the challenges that researchers face when using EMR data.

## 5. Clinical Guidelines & Outcome

Any discourse on “Precision Medicine in Solid Tumors” remains incomplete without presenting views on the clinical guidelines and outcomes which embody “response evaluation”. Historically, attempts to define the objective response of a tumor to an anticancer agent were made as early as the early 1960s [28]. Following the introduction of specific criteria for the codification of tumor response evaluation in the late 1970s by the International Union Against Cancer and the World Health Organization (the 1979 WHO Handbook), various organizations involved in clinical research reviewed these criteria in 1994 to ready a set of guidelines. Down the road, a model by which response rates could be derived from the unidimensional measurement of tumor lesions instead of the usual bi-dimensional approach was developed, which was validated by the Response Evaluation Criteria in Solid Tumors Group. The philosophic background to clarify the various purposes of “response evaluation” has been presented in an article by Patrick Therasse et al. [29]. The article covers several aspects of response evaluation, including: (1) details of methods of assessing codified tumor lesions within the guidelines; (2) Response Evaluation Criteria In Solid Tumors (RECIST) guidelines; (3) Response Outcomes in Daily Clinical Practice of Oncology; (4) Response Outcomes in Uncontrolled Trials as a Guide to Further Testing of a New Therapy; and (5) Response Outcomes in Clinical Trials as a Surrogate for Palliative Effect. With the advent and success of tumor immunotherapy, attempts have been made to define systematic criteria, designated immune-related response criteria, to include additional response patterns observed with ICI therapy beyond those described by Response Evaluation Criteria in Solid Tumors or WHO criteria, especially in advanced melanoma [30,31,32,33]. Among them, Wolchok et al. put forward novel criteria to better capture the response patterns observed with immunotherapies, “Immune-related Response Criteria” (irRC) [33]. The irRC has since then presented a more comprehensive evaluation of immunotherapies in clinical trials, in conjunction with either RECIST or WHO, proving that irRC is a powerful criterion for outcome measurement in clinical investigation. In a retrospective study, Kuroda et al. presented data on the clinical guideline-guided Outcome consistency for surgically resected stage III NSCLC, demonstrating that the guideline-consistent alternatives, which comprise ATSR (adjuvant treatments after surgical resection) or GMT-R (guideline-matched first-line treatment for recurrence), can contribute to survival benefits in pathological stage III NSCLC.

Today’s “Precision Medicine in Solid Tumors” is an evolution of medical practice in progress, a perfect example of the power of the interdisciplinary approach. It remains to see how the future liaison of classical medicine and translational research, equipped with technology, bioinformatics, data safety, advocacy, and social media, will shape the deliverance of patient care in medicine.

In this Special Issue, we tried an uphill task to present a scientific interrogation on salient critical features of “Precision Medicine in Solid Tumors.” We will consider ourselves immensely humble if our collected reviews on the specific topics are of help to our readers.
